# Modified subcutaneous lumbar spine index (MSLSI): a new predictor for early surgical site infection after transforaminal lumbar interbody fusion

**DOI:** 10.1080/07853890.2025.2597693

**Published:** 2025-12-09

**Authors:** Xu Shen, Chen Yao, Jiahua Duan, Zengxin Gao, Beiyue Wang

**Affiliations:** ^a^ Department of Joint Surgery, The Second Affiliated Hospital of Nanjing Medical University, Nanjing, China; ^b^ Department of Orthopedics, Zhongda Hospital, School of Medicine, Southeast University, Nanjing, China

**Keywords:** Modified subcutaneous lumbar spine index, lumbar interbody fusion, early surgical site infection, risk factor

## Abstract

**Purpose:**

The purpose of this study is to explore the value of Modified subcutaneous lumbar spine index (MSLSI) as a new predictor for early surgical site infection (SSI) after transforaminal lumbar interbody fusion (TLIF).

**Methods:**

We conducted a retrospective case–control study involving patients who underwent TLIF between 2015 and 2021 at our hospital. Thirty-one patients with early postoperative SSI were enrolled in cases group, and the controls were matched (1:2) using the following criteria: gender (female/male), age (±3 y), diabetes (insulin/oral/no), date of surgery (morning/afternoon) and season of surgery (spring/summer/autumn/winter). MSLSI was measured on mid-sagittal T2-weighted MRI imaging. Pearson correlation analysis was calculated to evaluate the relationship between MSLSI and BMI. The Receiver Operating Characteristic (ROC) curve was performed to evaluate the value of MSLSI and subcutaneous fat thickness in predicting the early SSI following TLIF. The radiographic parameters were measured independently by two doctors using the Picture Archiving Communication System (PACS).

**Results:**

Ninety-three patients were enrolled in final analysis, with 31 cases in SSI group and 62 matched controls. Univariate and multivariate logistic regression analysis indicated that SSI were significantly correlated with obesity (OR = 6.43, 95%CI = 1.16–35.73, *p* = 0.033), subcutaneous fat thickness (OR = 4.53, 95%CI =1.32–15.52, *p* = 0.016) and MSLSI (OR = 6.17, 95%CI = 1.73–22.12, *p* = 0.005). The ROC curve demonstrated that the risk of early SSI development increased when the MSLSI was ≥0.68. Pearson correlation analysis indicated that MSLSI was moderate correlation with body mass index (BMI).

**Conclusion:**

MSLSI represents an innovative radiographic index that independently predicts early SSI risk after TLIF.

## 1. Introduction

Surgical site infection (SSI) is a frequent complication following transforaminal lumbar interbody fusion (TLIF), which may increase medical expenditures, extend hospitalization and result in additional surgery [1–3]. The morbidity of SSI has been reported to be between 0.7% and 12% in primary lumbar fusion surgery [4,5]. Multiple studies have reported various risk factors for SSI following TLIF, including diabetes, obesity, multilevel surgery, longer surgery duration, drainage tube duration, subcutaneous fat thickness and SLSI [6–8].

Obesity (BMI ≥ 28kg/m^2^) is a well-described risk factor for SSI in TLIF surgery, but BMI is calculated based on weight and height alone and does not take into account the fat distribution characteristics [9,10]. Subsequent studies demonstrated that lumbar subcutaneous fat thickness had advantages compared to BMI [8,11–13]. Mehta et al. [14] reported 298 lumbar interbody fusion cases and indicated SSI was associated with lumbar subcutaneous fat thickness but not with BMI. However, Shaw [15] thought that subcutaneous fat thickness did not take into account the effect of spinous process height on the early SSI in TLIF surgery. Therefore, Shaw [15] introduced subcutaneous lumbar spine index (SLSI), defined as the ratio of subcutaneous fat thickness at the lumbar surgical site to the spinous process height of the corresponding vertebra. We previously reported that SLSI was a better predictor than subcutaneous fat thickness of the risk of early SSI in TLIF surgery [16]. However, the measurement of SLSI was cumbersome and poor reproducibility. Wang et al. [17] proposed a new concept of Modified subcutaneous lumbar spine index (MSLSI), which was defined as the ratio of subcutaneous fat thickness to the spinous process height at L4 level. MSLSI needed to be done only once on MRI mid-sagittal T2-weighted images, not only saved time, but also improved stability and accuracy of measurement. To our knowledge, no literature has yet reported the impact of MSLSI on early SSI following TLIF.

In our study, we conducted a retrospective case-control study to evaluate the value of MSLSI as a potential risk factor for SSI in TLIF surgery.

## 2. Materials and methods

### 2.1. Demographic data

All consecutive patients undergoing TLIF (including L4) at our institution between 2015 and 2021 were retrospectively reviewed. The exclusion criteria: (1) age ≤ 18 years; (2) spinal infection, tumours, spine malformation or revision surgery; (3) incomplete MRI and medical data. (4) minimally invasive fusion surgeries (MIS-TLIF). All cases signed informed consent before surgery. All the TLIF approaches were posterior, median longitudinal incision. Intravenous antibiotics were used to prevent infection 30 min before surgery and 24 h after surgery. Cephazolin was the first-choice antibiotic, clindamycin was selected as allergic to cefazolin. This study was approved by the institutional ethics committee of Zhongda Hospital (IRB:2023ZDSYLL400P01), and informed consent was obtained from all participants. The study had adhered to the principles stated in the ‘Declaration of Helsinki’.

### 2.2. Definition of SSI

**Figure 1.**
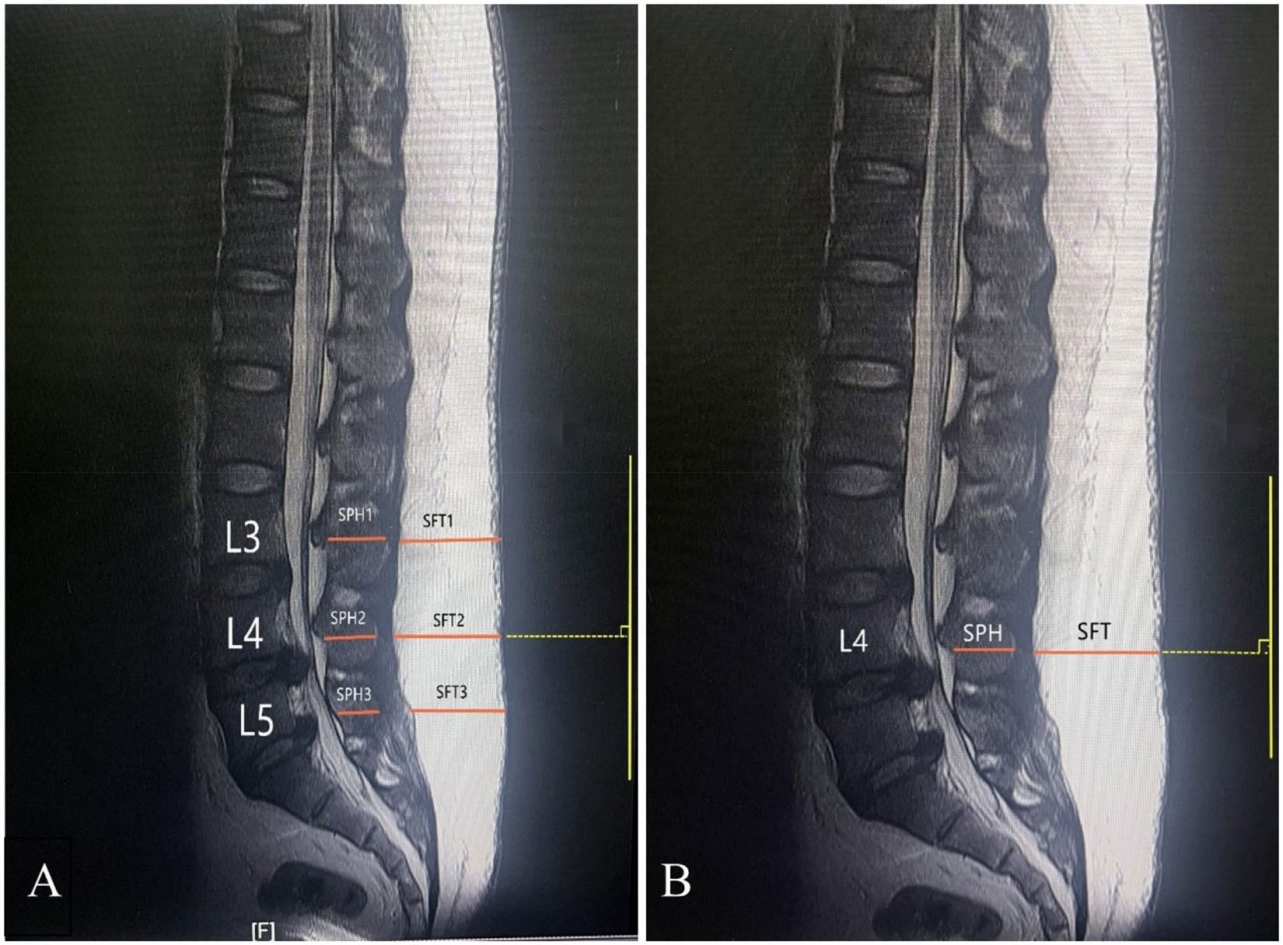
The measurements of lumbar subcutaneous fat thickness (SFT) and spinous process height (SPH) at the operated levels (excluding S1) on T2 midsagittal MRI. A: Traditional measurement: SLSI=(SFT1/SPH1 + SFT2/SPH2 + SFT3/SPH3)/3. B: New predictor: MSLSI: The measurements only at the level of L4.

Early SSI is defined as infection developing within 30 days after surgery according to the Centres of Disease Control (CDC) and Prevention definition [18]. Early SSI is divided into superficial infection and deep infection. Superficial SSI only involves skin and subcutaneous tissue and has one of the following conditions: (1) The incision has purulent secretion; (2) The incision secretions cultured bacteria; (3) The incision has pain or tenderness, swelling, redness; (4) Superficial SSI is diagnosed by the surgeon. Deep SSI is defined as an infection involving deep fascia and muscle and has one of the following conditions: (1) Pus flows out of the deep incision; (2) The deep incision splits spontaneously accompanying fever or pain. MSLSI was defined as the ratio of subcutaneous fat thickness to the spinous process height at L4 level on mid-sagittal T2-weighted MRI images (Figure 1).

### 2.4. Statistical analysis

Statistical analyses were performed using SPSS 20. Univariate and multivariate logistic regression analysis assessed the risk factors for early SSI. Pearson correlation analysis assessed the correlation between BMI and MSLSI. ROC curve analysis was performed to find optimal cut-off value of MSLSI. Statistical significance was defined as a *p* < 0.05.

## 3. Results

### 3.1. General demographic data

A total of 1425 cases were enrolled in our study, 31 of whom were identified early SSI (17 deep infections and 14 superficial infections). 62 control cases were selected according to the matched criteria. The general demographics between two groups were shown in Table 1.

**Table 1.** General demographic between cases and controls.

Variable	SSI group(*n* = 31)	Non-SSI group(*n* = 62)	*P*
Age (years)	60.55 ± 12.72	60.42 ± 12.35	0.96
Gender			
Male	17(54.8%)	33(53.2%)	0.89
Female	14(45.2%)	29(46.8%)	
Diabetes			
insulin	3(9.7%)	6(9.7%)	0.91
oral	4(12.9%)	9(14.5%)	
No	24(77.4%)	47(75.8%)	
timing of surgery			
Morning	14(45.2%)	28(45.2%)	1.00
Afternoon	17(54.8%)	34(54.8%)	
season of surgery			1.00
Spring	8(25.8%)	16(25.8%)	
Summer	6(19.4%)	12(19.4%)	
Autumn	10(32.2%)	20(32.2%)	
Winter	7(22.6%)	14(22.6%)	
Smoking			0.65
Yes	19(61.3%)	34(54.8%)	
No	12(38.7%)	28(45.2%)	
ASA grade			0.31
I	7(22.6%)	17(27.4%)	
II	16(51.6%)	37(59.7%)	
III	8(25.8%)	8(12.9%)	
Number of fusion levels			
1	8(25.8%)	23(37.1%)	0.15
2	15(48.4%)	30(48.4%)	
3	8(25.8%)	9(14.5%)	
Obesity, BMI ≥ 28kg/m^2^			0.003
Yes	15(48.4%)	12(19.4%)	
No	16(51.6%)	50(81.6%)	
BMI (kg/m^2^)	26.32 ± 3.28	25. 1 ± 2.8	0.07
Operation time (min)	167.1 ± 62.14	157.26 ± 47.22	0.40
The volume of drainage (ml)	501.61 ± 207.96	451.13 ± 185.58	0.24
Subcutaneous fat thickness (mm)	22.75 ± 4.61	19.42 ± 4.69	0.002
MSLSI	0.68 ± 0.22	0.51 ± 0.19	*p* < 0.001

The results indicated that there were no statistical differences in the baseline characteristics between two groups. However, the percentage of obesity was higher in the SSI group (48.4%) than controls (19.4%). Subcutaneous fat thickness (22.75 ± 4.61 mm), MSLSI (0.68 ± 0.22) in the SSI group were higher than controls (*p* < 0.05).

### 3.2. ROC curve analysis of MSLSI and subcutaneous fat thickness

**Figure 2.**
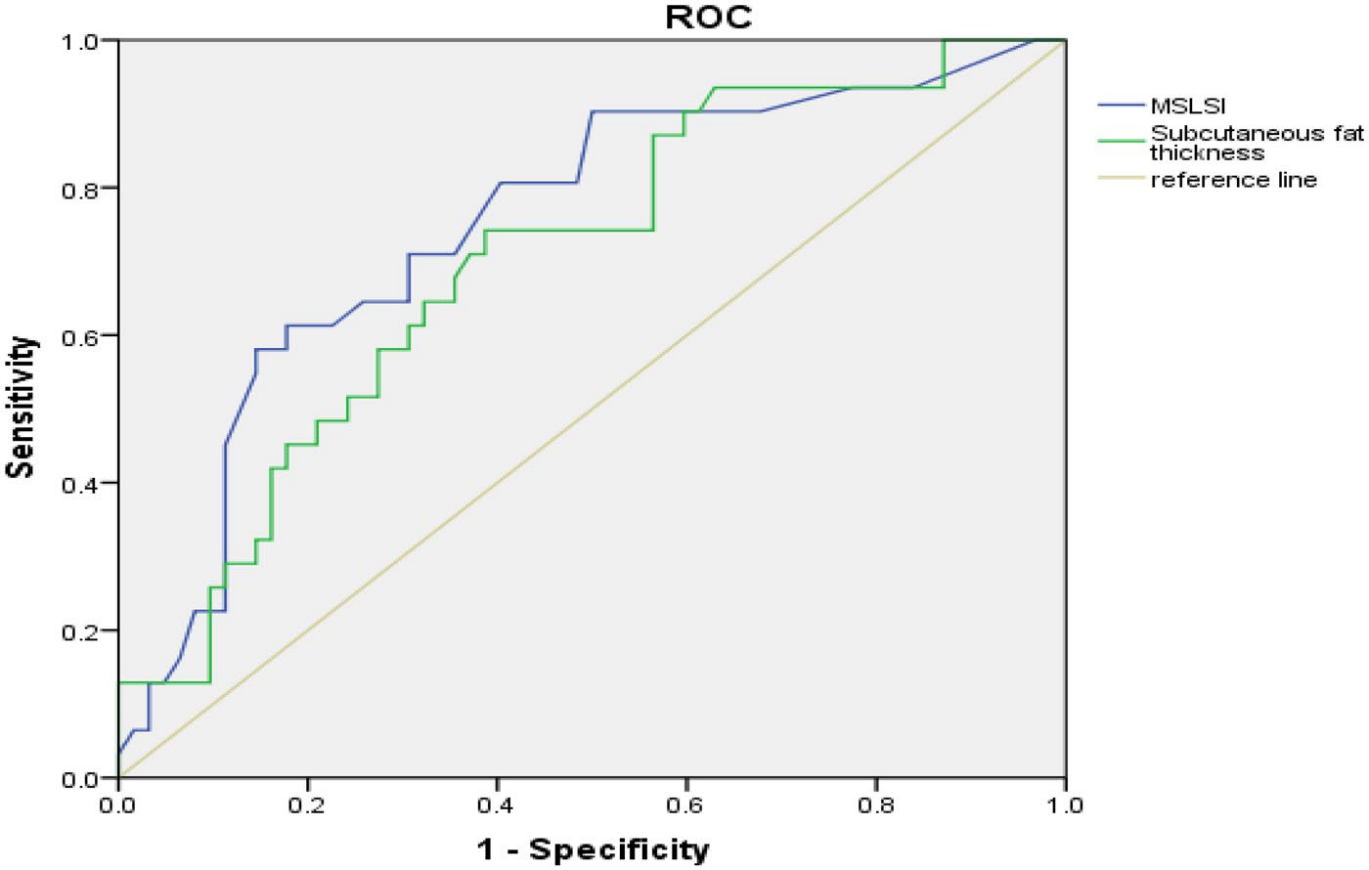
ROC curve of subcutaneous fat thickness and MSLSI to predict SSI after TLIF.

The ROC curve showed that lumbar SFT and MSLSI were the independent risk factors for early SSI (Figure 2). The area under the curve (AUC) of MSLSI was 0.749 (85.5% specificity and 58.1% sensitivity) and the cut-off was 0.68 (Youden index: 0.435), while the AUC of subcutaneous fat thickness was 0.701 (64.5% specificity and 71% sensitivity) and the cut-off was 20 (Youden index: 0.355).

### 3.3. Multivariate logistic analysis of risk factors for early SSI

**Figure 3.**
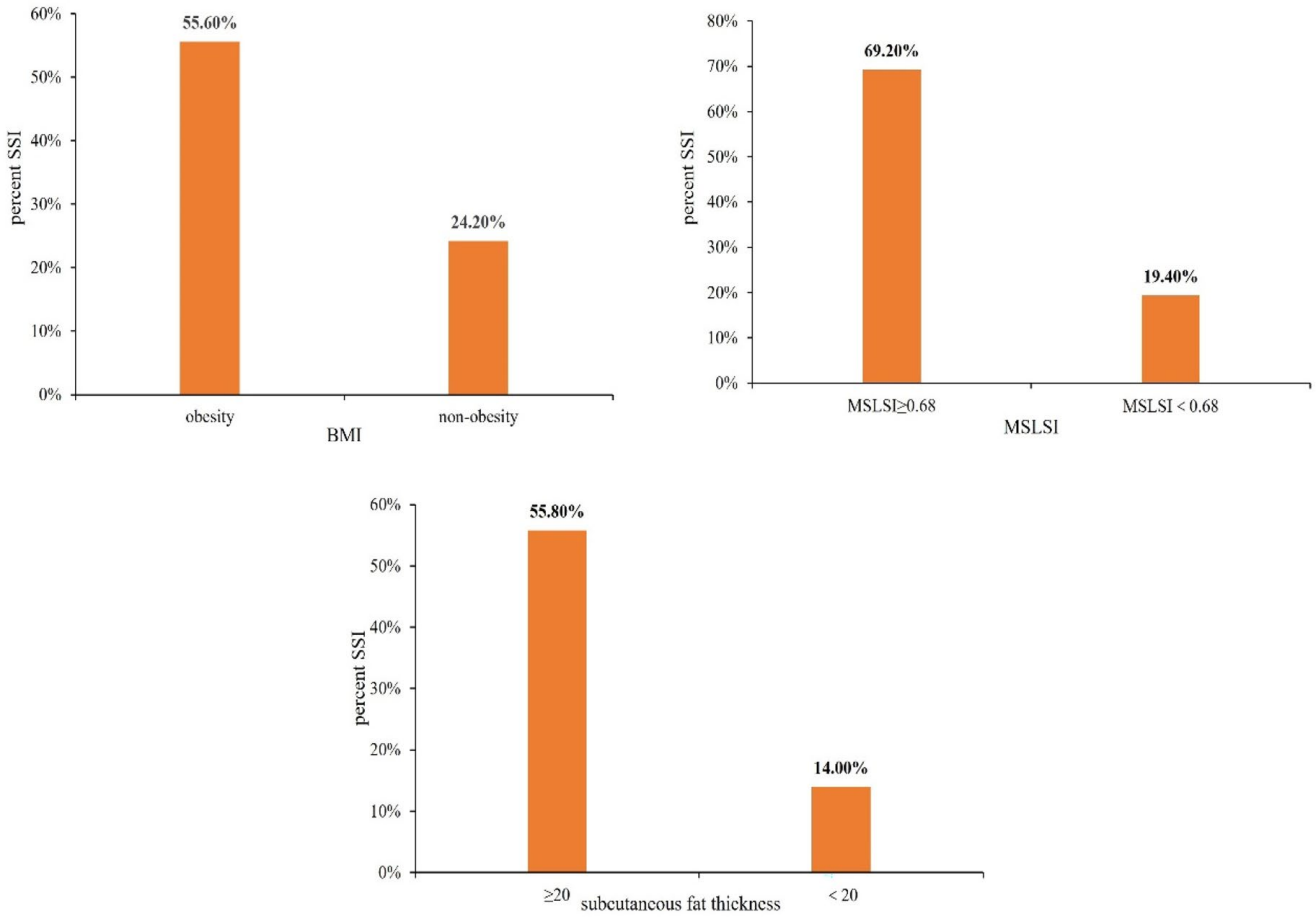
The prevalence of SSI according to obesity, MSLSI and subcutaneous fat thickness.

Multivariate logistic analysis showed obesity (OR = 6.43, 95%CI =1.16–35.73, *p* = 0.033), greater subcutaneous fat thickness (OR = 4.53, 95%CI =1.32–15.52, *p* = 0.016) and greater MSLSI (OR = 6.17, 95%CI = 1.73–22.12, *p* = 0.005) were independent risk factors for postoperative early SSI (Table 2). The percentage of SSI development was 55.6% (15 of 27) in obese people, 69.2% (18 of 26) in patients with MSLSI ≥0.68, 55.8% (24 of 43) in patients with subcutaneous fat thickness ≥20mm (Figure 3).

**Table 2.** Multivariate analysis.

Risk Factors	Odds Ratio (95% CI)	P
MSLSI, ≥0.68	6.17(1.73–22.12)	0.005
Subcutaneous fat thickness, ≥20mm	4.53(1.32–15.52)	0.016
Obesity	6.43(1.16–35.73)	0.033

### 3.4. Pearson regression analysis of the association of MSLSI with BMI

**Figure 4.**
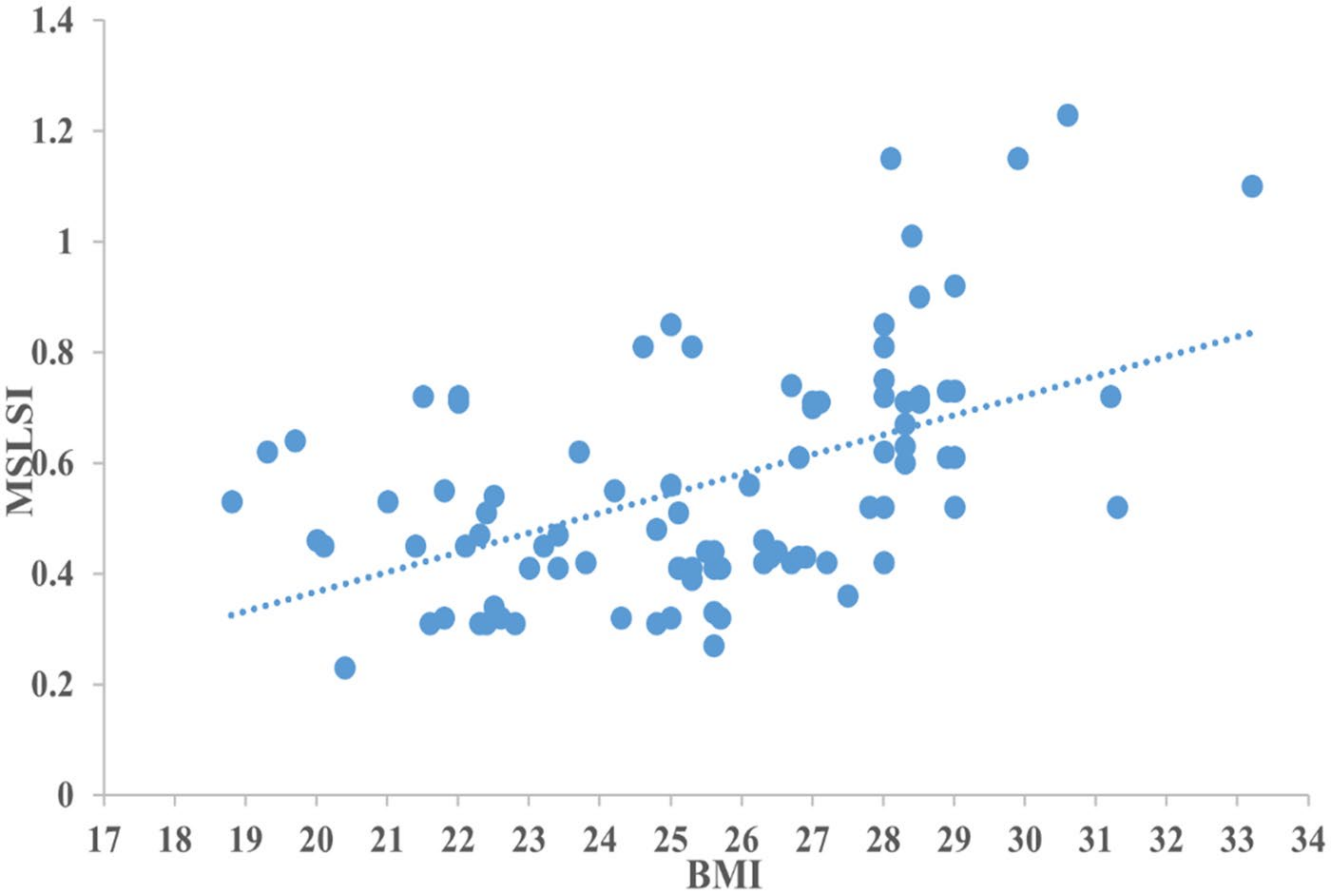
Pearson correlation analysis showed that MSLSI was positively correlated with BMI.

Pearson correlation analysis showed that MSLSI was positively correlated with BMI (Figure 4, *r* = 0.51, *p* < 0.001).

## 4. Discussion

Currently, the incidence rate of (LDD) has gradually increased, TLIF technique is commonly considered the gold standard for LDD with instability [19–21]. However, postoperative complications are increasing as TLIF becomes more globally popular, among them, early SSI is a relatively frequent complication, which can prolong antibiotic use, re-debridement and drainage, potential endograft removal, and the risk of bone non-union [22–24]. Prolonged hospital stays, additional medical interventions, and post-operative follow-up of relevant imaging and laboratory tests inevitably increase the cost of treatment, which can put tremendous financial pressure on patients [25,26]. Previous studies showed that risk factors for early SSI in TLIF include diabetes, obesity, length of operative time, multilevel fusion levels and subcutaneous fat thickness [1,3,9,12]. In previous article, we have demonstrated SLSI was a better predictor for SSI development after TLIF [16]. However, the measurement of SLSI is cumbersome, poor reproducibility and also carried out on multiple lumbar segments. Meanwhile, the measurement of fat thickness is greatly affected by the patient’s age, gender, height, BMI and other factors, which can result in great variation in practical applications [12]. Consequently, we performed a cohort study that demonstrated MSLSI as a significant predictor of early SSI.

In this study, multivariate logistic analysis revealed obesity, subcutaneous fat thickness and MSLSI were risk factors for early SSI following TLIF. Patients with MSLSI ≥0.68 who underwent TLIF procedure had a 3.6-fold increase in risk of SSI compared with those with MSLSI ≤0.68. Pearson correlation analysis indicated a moderate correlation between BMI and MSLSI (*r* = 0.51). Actually, BMI is calculated based on weight and height, it only reflects total body fat mass and does not consider lumbar fat distribution. Furthermore, BMI cannot distinguish between muscle and fat mass [10,27]. In our research, BMI was not an independent risk factor for early SSI following TILF procedure. This conclusion was consistent with previous studies.

During TLIF procedure, a higher MSLSI portended thicker subcutaneous fat or shorter spinous process, this increased in index always resulted in increasing incision length, extending surgical time and increasing tissue necrosis, which can increase the risk of infection of the surgical incision.

The ROC curve analysis demonstrated that the MSLSI and subcutaneous fat thickness exhibited AUC values of 0.749 and 0.701, respectively, indicating MSLSI and subcutaneous fat thickness may serve as valuable prognostic indicators for SSI in spinal surgery patients. These findings indicated the MSLSI was more sensitive than subcutaneous fat thickness at predicting risk of SSI following TILF surgery. This phenomenon may be attributed to the fact that even in patients with normal subcutaneous fat thickness, congenitally low-lying lumbar spinous processes can create intraoperative challenges in achieving adequate surgical exposure. Such anatomical constraints often necessitate excessive tissue manipulation and prolonged retraction, thereby increasing the risk of surgical infection [28,29].

This study has several noteworthy limitations. First, the number of SSI cases were relatively small. Second, other influential variables related to SSI were not included in the study. Third, we did not perform subgroup analyses for superficial and deep infections. Therefore, a multicenter prospective study with larger sample sizes is warranted to validate this result in the future.

## 6. Conclusion

MSLSI represents an innovative radiographic index that independently predicts early SSI risk after TLIF.

## Data Availability

The data that support the findings of this study are available from the corresponding author upon reasonable request.
